# Cellular Innate Immunity against PRRSV and Swine Influenza Viruses

**DOI:** 10.3390/vetsci6010026

**Published:** 2019-03-11

**Authors:** Elisa Crisci, Lorenzo Fraile, Maria Montoya

**Affiliations:** 1 Department of Population Health and Pathobiology, College of Veterinary Medicine, North Carolina State University, Raleigh, NC 27607, USA; ecrisci@ncsu.edu; 2 Comparative Medicine Institute, North Carolina State University, Raleigh, NC 27607, USA; 3 Universitat de Lleida, 25198 Lleida, Spain; lorenzo.fraile@ca.udl.cat; 4 Centro de Investigaciones Biológicas, Consejo Superior de Investigaciones Científicas (CIB-CSIC), 28040 Madrid, Spain

**Keywords:** pig, innate immunity, PRRSV, swine influenza virus

## Abstract

Porcine respiratory disease complex (PRDC) is a polymicrobial syndrome that results from a combination of infectious agents, such as environmental stressors, population size, management strategies, age, and genetics. PRDC results in reduced performance as well as increased mortality rates and production costs in the pig industry worldwide. This review focuses on the interactions of two enveloped RNA viruses—porcine reproductive and respiratory syndrome virus (PRRSV) and swine influenza virus (SwIV)—as major etiological agents that contribute to PRDC within the porcine cellular innate immunity during infection. The innate immune system of the porcine lung includes alveolar and parenchymal/interstitial macrophages, neutrophils (PMN), conventional dendritic cells (DC) and plasmacytoid DC, natural killer cells, and γδ T cells, thus the in vitro and in vivo interactions between those cells and PRRSV and SwIV are reviewed. Likewise, the few studies regarding PRRSV-SwIV co-infection are illustrated together with the different modulation mechanisms that are induced by the two viruses. Alterations in responses by natural killer (NK), PMN, or γδ T cells have not received much attention within the scientific community as their counterpart antigen-presenting cells and there are numerous gaps in the knowledge regarding the role of those cells in both infections. This review will help in paving the way for future directions in PRRSV and SwIV research and enhancing the understanding of the innate mechanisms that are involved during infection with these viruses.

## 1. The Porcine Respiratory Complex: General Features and PRRSV and SwIV Involvement

The term porcine respiratory disease complex (PRDC) was used in the past to describe the pneumonia of multiple etiologies that cause clinical disease with negative consequences on productive parameters during the finishing process. Nowadays, the term delineates a more general term describing a polymicrobial syndrome that results from a combination of infectious agents, environmental stressors, population size, management strategies, age, and genetics that causes reduced performance, together with increase mortality rates and production costs in the pig industry worldwide. The etiology of the PRDC has been in continuous progression due to pathogen evolution as well as in management and stressor changes in pig farming [1,2,3].

The respiratory disease complex is the consequence of impairment of the normal respiratory immune system due to pathogens that are able to harm these defenses and establish infection on their own. Those microorganisms are normally considered to be the primary etiological agents and only, subsequently, other opportunistic agents appear in order to take advantage of the virulence mechanisms of the primary ones [1,2,3].

Porcine primary agents include viruses, like porcine reproductive and respiratory syndrome virus (PRRSV), swine influenza virus (SwIV), porcine circovirus type 2, pseudorabies virus, and bacteria, like *Mycoplasma hyopneumoniae*, *Bordetella bronchiseptica*, and *Actinobacillus pleuropneumoniae* [4,5,6]. Minor viral pathogens that are associated with respiratory implication are also the result of *paramyxoviridae* family viruses (such as porcine rubulavirus and Nipah virus), porcine cytomegalovirus, porcine respiratory coronavirus, porcine parvovirus, and porcine torque tenovirus [7]. *Pasteurella multocida*, *Haemophilus parasuis*, *Streptococcus suis*, and *Trueperella pyogenes* are other common minor bacterial agents that are only linked with respiratory manifestations [8], although this latter one can also cause primary respiratory disease, likely through a blood-borne route [1,2,3]. 

In this review, we will focus, in particular, on two enveloped RNA viruses, PRRSV and SwIV, as major etiological agents that contribute to PRDC and on the recent discoveries in porcine cellular innate immunity during PRRSV and/or SwIV infection.

### 1.1. Porcine Reproductive and Respiratory Syndrome Virus

Porcine reproductive and respiratory syndrome virus (PRRSV) is a member of the family *Arteriviridae*, which also includes the simian hemorrhagic fever virus (SHFV) and equine arterivirus (EAV). It is an enveloped, positive-stranded RNA virus and the viral genome, packed by nucleocapsid proteins, contains 11 known open reading frames (ORFs). The replicase gene consists in ORFs 1a and 1b regions that encode two large nonstructural polyproteins, pp1a and pp1ab, which are processed into at least 14 non-structural proteins (nsps). The other genes, ORF2-7, encode for four membrane-associated glycoproteins (GP2a, GP3, GP4, and GP5), three unglycosylated membrane proteins (E, ORF5a, and M), and a nucleaocapsid protein (N) [9,10]. PRRSV exists as two species, type 1 (European origin, PRRSV-1) and type 2 (North American origin, PRRSV-2), which share only 55–70% nucleotide identity, but its replication and recombination properties have led to the extraordinary phenotype and genotype diversity worldwide [11,12].

Swine are the only natural host of PRRSV and the virus has a very restricted tropism for cell of the monocytic lineage. The fully differentiated porcine alveolar macrophages have been considered as the cell target for PRRSV [13], but more recently parenchyma macrophage-like/pulmonary intravascular macrophages (PIM) have been described as also supporting PRRSV replication [14]. 

On the macrophages, CD163 has been determined to be the major receptor that mediates viral internalization and disassembly. CD169 (sialoadhesin or siglec-1) can also serve as a virus receptor via interaction with GP5/M ectodomains, but it is considered to be not essential for attachment and/or internalization. Other cellular receptors are heparan sulphate, vimentin, CD151, DC-SIGN (CD209), and, lately, siglec-10, has been shown to be involved in the entry process of PRRSV [15]. Recently, CD163 has been edited in pig zygotes using CRISPR/Cas9, so as to remove the viral interaction domain while maintaining protein expression and biological function. These “edited” pigs were resistant to PRRSV-1 as well as PRRSV-2 infection in vitro. Moreover, when these animals were challenged with a highly virulent PRRSV-1 subtype 2 strain, the edited pigs showed no signs of infection or viremia or antibody response indicative of a productive infection, which is in contrast to the wild-type control group. Thus, “edited” pigs are fully resistant to infection by PRRSV and confirm CD163 as major PRRSV receptor [16,17].

### 1.2. Swine Influenza Virus

Influenza viruses (IVs) are enveloped, single stranded RNA viruses belonging to the family *Orthomyxoviridae*. This family comprises different genera; in particular A, B, and C. Influenza A viruses are further classified into subtypes based on the antigenicity of their hemagglutinin (HA) and neuraminidase (NA) molecules. Currently, 18 HA (H1-H18) and 11 NA subtypes (N1-N11) have been described [18,19].

Influenza A and B viruses possess the segmented genome of eight single-stranded negative-sense RNA molecules that typically encode 11 or 12 viral proteins [20]. The viral envelope consists of a lipid bilayer that contains transmembrane proteins on the outside and matrix protein (M1) on the inside. The lipids that compose the envelope derived from the host plasma membrane and are selectively enriched in cholesterol and glycosphingolipids. The three transmembrane envelope proteins—HA, NA, and M2 (ion channel)—are anchored in the lipid bilayer of the viral envelope. HA is the major envelope protein forming the spikes [21]. HA provides the receptor-binding site and elicits neutralizing antibodies. It binds to a host cell receptor that contains terminal α-2,6-linked or α-2,3-linked sialic acid (α-2,6-SA or α-2,3-SA) moieties.

IVs infect different animal species and pigs (*Sus scrofa*) and they are one of the natural hosts of these viruses. Swine influenza is a highly important respiratory swine disease and the main causative viruses are type A IVs H1N1, H1N2, and H3N2 subtypes [22], which are antigenically related to human IVs. Pigs are susceptible to infection with avian and human IVs [23,24] and they are supposed to play an important role in human influenza ecology. In fact, genetic reassortment between human and/or avian and/or swine IVs can occur. The potential to generate novel IVs has resulted in swine being labelled “mixing vessels”. There are three facts that support the mixing vessel hypothesis: (1) swine are susceptible to avian and human viruses; (2) reassortment of swine/avian/human viruses occurs in pigs; and, (3) pigs can transmit reassortant IVs to humans [25]. Infection in pigs with IVs results in an acute respiratory infection that resolves within a week if no other complications are present with the activation of the immune system [26].

## 2. Porcine Innate Immune System

### 2.1. Dendritic Cells

Dendritic cells (DC) are key antigen presenting cells that prime naïve T cells and drive the adaptive immune response. They are very effective in sensing viruses through a wide range of surface, cytosolic, and endosomal receptors, and Toll-like receptors (TLR) are crucial DC pattern recognition receptors between those. 

Summerfield and McCullough firstly reviewed the Porcine DC family in 2009. In vitro, DC can be divided in monocyte-derived DC (moDC) and bone marrow derived DC (BMDC, considered conventional DC). In vivo, besides moDC and the conventional DC (cDC), we also have the plasmacytoid DC (pDC) and both are located in the blood and the mucosa of the different biological tracts. 

In vitro, moDC and BMDC generally express CD172a, CD1, CD14 low/+, CD16, SLAII +/hi, and CD80/86, while blood cDC normally present CD14−, low CD172a, and variable CD1 and CD16 expressions (Table 1). In all cases, cDC are CD4− and they predominantly express TRL2, TLR4, and TLR3 and TLR8. On the other side, pDC are generally CD172a low/−, CD123+, CD135+, CD1+/−, CD16+/−, CD4+, SLAII+, CD80/86low, and CD14− (Table 1), and they predominantly express TLR7 and TLR9 [27,28]. moDC have been extensively used in the last decade as an in vitro strategy to study the effect of different viruses on pivotal cells driving adaptive immunity. Generally, moDC have been generated after PBMC isolation, CD14+ selection, or PBMC plastic culture to isolate adherent monocytes. Using different amounts of GMCSF and IL4, moDC were harvested after 5–7 days of culture and immature or mature moDC were used for PRRSV infection [29]. 

In vivo, cDC have been mainly studied from peripheral blood mononuclear cells (PBMC) following different sorting strategies, given the low percentage of these cells in a normal pig (between 0.1–1%). Their phenotype is as stated above, but other markers have been used to define different subsets as cDC1 or cDC2 using CD1, CADM1, or XCR1 [30]. Mucosal DC present a similar basic phenotype to cDC and pDC, but the surface marker expressions showed different profiles, depending on the biological tract considered. Pulmonary and tracheal DC have been characterized into three distinctive populations according to their phenotype and functional capacities: cDC1, cDC2, and inflammatory DC [31]. Given the respiratory tropism of PRRSV and SwIV, in this review we will only focus on results that were obtained using in vitro derived-DC and primary DC present in the respiratory tract.

### 2.2. Macrophages

Some macrophage (MΦ) precursors differentiate in the bone marrow into monocytes, which enter the blood stream. They then migrate to the different tissues, where they further differentiate into specific macrophages. They constitute the so-called mononuclear phagocyte system (MPS). MΦ are considered to be antigen presenting cells and they have important regulatory and effector functions in the specific immune response and in the maintenance of tissue homeostasis [32].

Two MΦ subsets are recognized, being referred to as M1 and M2, which result from classical or alternative activation, respectively. Classical (M1) activation of MΦ requires two signals, namely IFNγ and TLR ligation, and they can be generated in vitro using IFNγ and LPS. M1 macrophages are able to kill intracellular pathogens infecting them, and then produce pro-inflammatory cytokines, including IL1β, TNFα, IL6, IL12, and IL23. Alternative (M2) activation of macrophages occurs via IL4 or IL13 and reflects increased mannose receptor expression (CD206) and are distinct from M1 MΦs by their limited killing ability. M2 MΦs are associated with wound repair, producing components for extracellular matrix synthesis [33,34,35].

Porcine macrophages express CD163, a scavenger receptor, CD169 (also known as sialoadhesin or siglec-1), and SWC9/CD203a (found in lung macrophages). Additionally, the other monocytic lineage markers are CD172a, CD14, CD16, and SLAII. They mainly express TLR2, TLR4, and TLR3, 7, 8 [32]. Porcine lung macrophages can be divided based on the microenvironment within the lung: alveolar macrophages (AM), pulmonary intravascular macrophages (PIM) and interstitial macrophages (IM) [36,37]. Swine PIM and IM have been recently included in the so-called AM-like macrophages [14].

### 2.3. Neutrophils

Neutrophils or polymorphonuclear neutrophils (PMNs) are the first line of specialized innate phagocytes during acute pathogens infection. They are an important component in the simulation of the inflammatory response, in some cases, causing detrimental collateral damage and killing pathogens through phagocytosis, degranulation, and extracellular traps (NETs) formation. During the NETs formation they release antimicrobial peptides, such as several neutrophil serine proteases (NSPs). Swine PMNs are SWC1+ or CD21+ and SWC8+ display the same morphology as those of humans, but are a smaller size, with lower granularity and higher activation threshold [27,38].

### 2.4. Natural Killer Cells

Circulating porcine lymphocyte population features are unusual when compared with human and mice populations, since they present abundant natural killer (NK) and γδ T cells [39,40]. NKT cells are not included in this section.

NK cells are a component of the innate immune system with the ability to spontaneously attack pathogen-infected and malignant body cells as well as to produce regulatory cytokines, such as IFNγ. They lyse virus-infected target cells and up regulate effector/activation molecules, like perforin and CD25. In some cases of activation, an additional SLAII DR expression was described. Porcine NK cells have been identified by a complex phenotype of perforin+, CD2+, CD8α+, CD8β-, CD11b+, CD16+, CD3−, CD4−, CD5−, CD6− [39,41,42,43], and for the expression of NKp46, an evolutionary conserved mammal receptor that belongs to the family of natural cytotoxicity receptors (NCRs). Studies considering NKp46 expression in pigs defined three distinct NK-cell subsets: NKp46−, NKp46+, and NKp46hi CD3− lymphocytes that display the phenotypic and functional properties of NK cells [42,43]. A distinct population of CD3+NKp46+ cells could also be identified where the majority of CD3+NKp46+ cells express CD8αβ heterodimer, comparable to porcine cytolytic T cells, while a minor subset belongs to the TCRγδ+ T cells. Nonetheless, the CD3+NKp46+ cells express NK-associated molecules, such as perforin, CD16, NKp30, and NKp44. Functionally, they respond to in vitro stimulation in a NK-like manner and they have the capacity of spontaneous cytolytic activity. Degranulation could be induced in CD3+NKp46+ lymphocytes by receptor triggering of both NKp46 and CD3 [42,43].

### 2.5. γδ T Cells

Swine, together with ruminants and birds, belongs to the group of γδ high species in which γδ T cells are not preferentially limited to epithelia and they may account for 25–85%, depending on the age of all circulating peripheral blood lymphocytes (PBL). T cells of γδ lineage are evolutionary conserved cells that develop in the thymus similarly to αβ T cells, but they do not need any selection for pre-antigen receptors and therefore mature faster than αβ T cells. γδ T share many features with αβ T cells, such as potent cytotoxic activity, regulatory functions, including the ability to induce maturation of dendritic cell, the capacity to produce a variety of cytokines, and they also generate and retain immunologic memory. γδ T respond rapidly to infection and they are probably involved mainly in mucosal immunity. They can act as antigen-presenting cells and their TCR recognizes a broad spectrum of unprocessed or non-peptide antigens without any requirement for MHC co-signalization. Due to their nature, the γδ T cells are often categorized as unconventional T cells and probably form a unique link between innate and adaptive immune responses [40,41,44,45].

Traditionally, γδ T cells in swine are subdivided into three subsets based on their expression of CD2 and CD8 and they include CD2−CD8−, CD2+CD8−, and CD2+CD8+ cells. These individual subsets differ in their homing characteristic and cytotoxic activities. Porcine γδ T cells have two levels of TCRγδ expression: TCRγδmed cells are mostly CD3+CD2+CD8− and CD2+CD8+, whereas TCRγδhi cells are highly enriched for CD2−CD8−. Finally, many γδ T cells can constitutively express CD25 and MHCII and the frequency of γδ T cells that are positive for CD25, CD11b, SWC1, and SWC7 can be increased by stimulation [40,41,44,45].

## 3. Innate Cellular Immune Responses Triggered by PRRSV

PRRSV is capable of causing reproductive and respiratory disease, and PRRS has an estimated annual cost to the swine industry of 664 million dollars in the United States of America (USA) [46]. The innate immune system is the first line of defense against any infection and, in particular, for PRRSV, lung MΦ, and DC is critical in the prevention of viral invasion in the blood circulation and inducing protective adaptive immunity.

Generally, PRRSV elicits poor innate responses that are associated with incomplete viral clearance in most of the pigs, depending on their age and immune status [9,10]. Infection with certain PRRSV strains induces significant suppression of NK cytotoxic activity and the quantity of the innate cytokines secreted in PRRSV-infected pigs is significantly lower than other viral infections and it is strain dependent [9,10,47]. PRRSV infection is generally a poor inducer of type I IFNs and its level remains low throughout the course of infection, as noted in pigs that were infected with many field isolates. Thus, to establish clinical disease in pigs, PRRSV modulates the host innate immunity through the dysregulation of NK cell function and IFNs production [34]. A recent study has provided new insight by showing how new virulent strains can differently modulate the inflammatory response toward a Th1 response in the lung [48].

### 3.1. Macrophages

Porcine alveolar macrophages (PAMs) have been extensively studied during PRRSV infection as the primary cell target of the virus. In this section, we will only review the latest discoveries while focusing on recent advances that were achieved with genomics approaches in primary PAMs and in the new insights on lung interstitial macrophages. When compared to porcine AM, PIM are equally permissive to PRRSV infection [14,37]. Almost two decades ago, PRRSV-2 antigens and nucleic acids have been demonstrated in PIM both in vitro and in vivo [37]. Examination of cultured PIM infected with PRRSV revealed the accumulation of viral particles in vesicles and the infection induces either PIM apoptosis or cell lysis. The PIM in vitro bactericidal activity is decreased as the in vivo phagocytic activity, measured by pulmonary copper clearance in PRRSV-infected pigs [37]. Recently, AM-like cells have been defined as macrophages phagocytosing blood-borne particles, which is in agreement with the PIM identity [14]. PIM were described as the major producer of PRRSV-1 Lena virus and their infection correlated better with viremia in vivo than AM infection. Thus, AM-like cells were as permissive as AM to PRRSV infection in vitro and in vivo, and PIM-expressed genes were characteristic of an embryonic monocyte-derived macrophage population [14].

Macrophages that were infected with PRRSV are functionally compromised in many ways, including cytokine production [34,49] and polarization [50]. PRRSV-2 prototype virus VR-2332 is one of the most studied strains, and the reactomes of infected PAM have been described at different time points. 573 differentially expressed genes (DEGs) were assigned into six biological systems, 60 functional categories and 504 pathways. Cell growth and death, transcription processes, signal transductions, energy metabolism, immune system, and infectious diseases formed the major reactomes of PAMs responding to PRRSV infection [51].

Only recently, data using different PRRSV-2 isolates suggests that macrophages polarization modulates PRRSV infection. Anti-viral cytokine expression was significantly higher in M1 macrophages than in M2 macrophages or non-polarized controls and both highly pathogenic (HP)(HuN4) and classic PRRSV (CH-1a) replication was significantly impaired in M1 PAMs [50]. Additionally, in HP PRRSV PAM infection (JXwn06), genes that are involved in IFN-related signaling pathways, pro-inflammatory cytokines and chemokines, phagocytosis, and antigen presentation and processing were significantly downregulated, indicating the aberrant function of PAM during the infection [52]. In particular, during early HP infection, the IFNβ downregulation seems to be mediated by a post-transcriptional inhibition through cellular miRNAs upregulation. This inhibition is stronger in HP when compared to a low pathogenic (LP) strain [53]. Additionally, lncRNAs have been reported during PRRSV and, in particular, 299 novel lncRNAs were differentially expressed after 12–24 hpi. All of the lncRNAs were enriched in pathways related to viral infection and immune response, particularly lncRNA TCONS_00054158 was adjacent to the TRAIL gene that was involved in apoptosis induction [54]. Moreover, during early infection, PRRSV-2 has been reported to induce both IL1β mRNA expression and secretion in a time- and dose-dependent manner, as mediated by the TLR4/MyD88 pathway and by the NLRP3 pathway [55,56]. The inhibitory effect appeared only in the late infection, where levels of pro-IL1β and procaspase-1 mRNA and the mature IL1β protein decreased to mock level. An IL1β antagonist, nsp11, and its endoribonuclease activity, encoded by the virus to limit antiviral reponses, mediated the effected [55].

Transcriptome differences between breeds during high pathogenic PRRSV infection have also been highlighted. Previous studies showed that Large White (LW) breed are more susceptible to PRRSV than Chinese breed Tongcheng (TC). At 7 dpi, PRRSV-infected PAM from TC showed 1257 differentially expressed genes (DEGs) involved in hepatic fibrosis/hepatic stellate cell activation, phospholipase C, granulocyte adhesion, and diapedesis pathways. In particular, 549 specific DEGs, including VAV2, BCL2, and BAX, were enriched in activation of leukocyte extravasation and suppression of apoptosis. On the other hand, 898 specific DEGs were defined in LW pigs, including genes that are involved in the suppression of Gαq and PI3K-AKT signaling. In this study, the authors proposed that in TC, the promotion of extravasation, migration of leukocyte and suppression of apoptosis constitute the defense mechanism against PRRSV [57].

**Figure 1:**
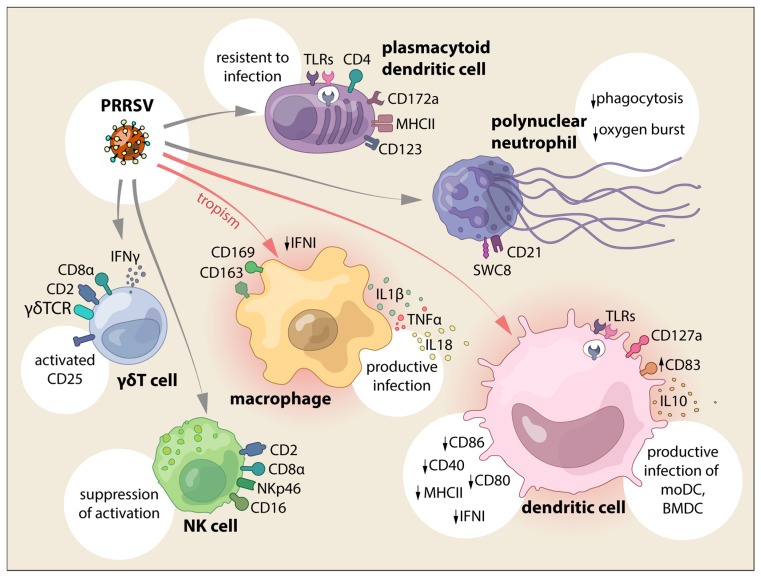
PRRSV interaction with cells from the innate immune system and the main effects reported.

**Figure 2:**
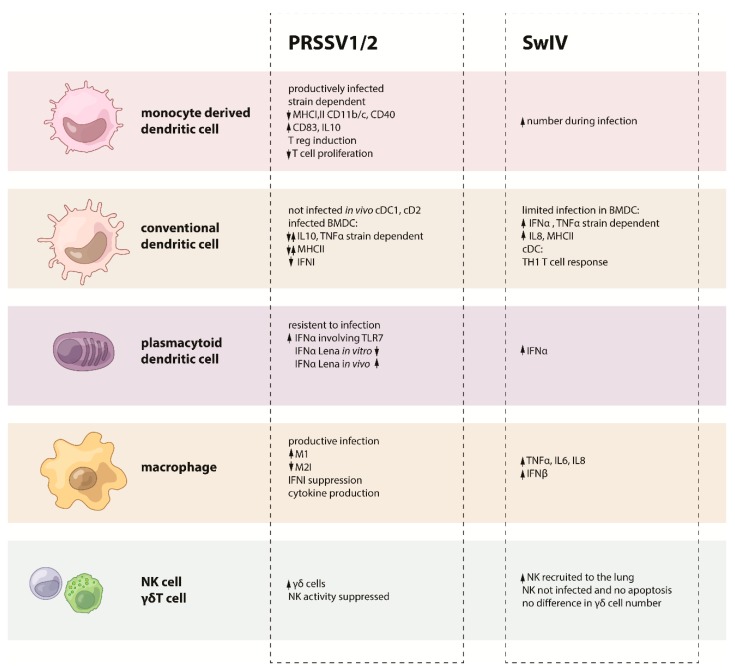
Summary on the PRRSV or SwIV effects on each cell from the innate immune system.

In summary, an aberrant antiviral response is induced in PAMs by PRRSV infection, suppressing IFN type I induction, and M1 polarization impair viral replication (Figure 1 and Figure 2).

### 3.2. Dendritic Cells

The PRRSV infection of DC has been controversial in the last decade, which is mainly due to the different in vitro DC generation systems and the intrinsic variability of the virus. In this section, we will summarize the most common DC systems that are used when infecting with PRRSV, the most relevant discoveries, and the new knowledge in the field.

#### 3.2.1. Conventional DC and Monocyte-Derived DC

Several PRRSV-1 and 2 viruses have been used when infecting DC, showing how the different outcomes are also related to the different strains and multiplicity of infection (MOI) used in the in vitro system. All in all, there is a clear agreement in the scientific community regarding the ability of PRRSV to productively infect moDC in vitro, despite the kinetics divergences that are related to the different MOI and time employed [58,59,60,61,62,63] and the variance between immature and mature moDC [64] or the proliferation levels of certain PRRSV strain [65].

Inconsistent data regarding PRRSV infection of other DC subsets are noticeable when primary lung and tracheal DC are considered. In fact, a recent publication [66] using primary lung DC, which was generated with enzymatic treatment and with an unclear DC phenotype (Table 1), showed that PRRSV-1 Lelystad (LV) virus was able to infect lung DC more efficiently in Duroc than in the Pietrain breed. On the other side, starting with Loving et al. in 2007 and finishing with recent results from both Resendiz et al. and Bordet et al. in 2018, it has been demonstrated that primary lung and tracheal DC are unable to support PRRSV-1 and 2 replication [48,60,67]. In Loving et al., lung DC were generated by density gradient separation and the selection of CD11c+ (Table 1), and PRRSV-2 NADC-8 did not infect them [60]. In Bordet et al., PRRSV-1 LV, Flanders13 and Lena did not infect, in vivo or in vitro, lung cDC1 cDC2 and moDC (only some residual infection in moDC) (Table 1) [48]. Following the same line of results, tracheal cDC1 and cDC2 (Table 1) were not susceptible to PRRSV-2 CIAD008 [67]. The scenario exhibits another layer of complexity when considering surface markers expression and T cell proliferation in studies where the outcomes differ when considering the PRRSV genotype, strains, and time points that are used in each experimental system.

PRRSV-2 generally reduced the antigen presenting functions of moDC by downregulating the expression of SLAI, SLAII, CD14, and CD11b/c, and by impairing their ability to activate both allogeneic and syngeneic T cell proliferation (PRRSV-2 SD-23983) [68]. Similar results were obtained with CNV-3 [69] and NVSL 97-7895 [59]. Liaoning et al. show unchanged SLAII but decreased SLAI, CD40, and CD80 [65], and other Chinese high and low pathogenic viruses that modulated CD83 [70,71]. DC-SIGN was not shown to be relevant during PRRSV-2 infection of moDC [61], and several PRRSV-2 isolates and commercial Ingelvac PRRS MLV vaccine showed no reduction in levels of T CD25+ or IFNγ+ or TNFα+ cells that were cultured with infected moDC. In primary lung, DC PRRSV-2 infection does not modify CD80/86 expression but downregulates SLAI, whereas PRRSV infected lung DC conserved their normal T proliferation ability [60].

When PRRSV-1 was used in the experiments, some strains reduce SLAI and increase T proliferation without the production of IFNγ in T cells [63] and others increase SLAII and CD80/86 in PRRSV N+ when compared with N− cells [62]. Prior infection moDC showed high expression levels of CD163 and low CD169 and replication was clearly restricted to a CD163+ CD169dim phenotype. There were no differences in the proliferation and frequency of Foxp3 after co-culturing with infected moDC [62]. PRRSV-1 LV, FL13, or Lena showed no differences in CD80/86, CD40, SLAII, SLAI expression on primary lung DC [48], and moDC showed different infection phenotype during dexamethasone and IL10 treatment [64].

Taking into account all of this variability, Rodríguez-Gómez et al. [62] considered the problem of the moDC markers expression, explaining the divergent outcomes to the use of different viral strains and virulence in the same genotype and to a suboptimal characterization of the experimental protocol that were used to generate moDC.

The production of cytokines is the most diverse aspect of infection. Strains, time points, and in vitro conditions showed disparate results, from no mRNA IL10 change [58] to high IL10 production [59,63,67,69] during PRRSV infection. Some strains do not promote the Th1 response [48,68] (PRRSV-2), whereas others do [48,61,69]. Additionally, disparity is found when considering antiviral factors, on one side moDC and lung DC responded to PRRSV with increased transcription of IFNβ, but there were no alterations in IFNα, MX, or PKR transcripts [60], and on the other side PRRSV infection activated IFNα, IFNβ transcription, but block of IFNα production [72]. In this last case, PRRSV-2 efficiently activated IFNα/β transcription in moDC in a time-dependent and transient manner, but little or no detectable IFNα was found in the supernatant and cell lysate of infected PRRSV DC. This effect was shown to be PI3K activation-dependent, but the post-transcriptional mechanism blocking IFNα production is still undefined [72].

An important and surprising finding was the role of Foxp3 Tregs during PRRSV infection. Hernendez group [59,63,73] added several pieces of the puzzle, which introduced a new role of IL10 and Tregs during PRRSV infection. In particular, first using PRRSV-2 NVSL 97-7895 [59] and then other European strains (2992, 2993) [63], they showed increased IL10 production during infection. An important outcome was the lack of Treg induction by PRRSV-1 infected moDC [63]. On the other side, with PRRSV-2 NVSL 97-7895 and CIAD008, they showed the induction of Foxp3+ CD25+ cells in PRRSV infected DCs, reversible by IFNα treatment, and upregulation of TGFβ expression in co-culture, but not IL10. Additionally, the upregulation of Foxp3 mRNA and the suppressor activity of Tregs on PHA stimulated lymphocytes were shown [73].

Finally, more recent studies on the interaction between DC and PRRSV have been focused on intracellular pathways and transcriptome. Chen et al. showed the involvement of different viral proteins on CD83 expression. CD83 is induced and viral proteins (N, nsp1, nsp10) affect the CD83 promoter in a time and dose dependent manner via the NFkB and Sp1 signaling pathways. PRRSV stimulates the expression of Sp1 and NFkB mRNA and NSp1α impairs moDC function releasing soluble CD83. PRRSV infection inhibits TAP1 and ERp57 expression (MHC complex proteins) by the induction of soluble CD83 and an impaired ability to stimulate T proliferation [70,71].

On the other hand, Proll et al. performed the first Gene Ontology (GO) analysis to determine the immune response to PRRSV LV infection in lung DC of two different breeds (Duroc and Pietrain). Although the phenotyping of DC was not very specific and lung DC probably included a macrophage component, the transcriptome profile showed breed specific differences in response to the infection. They identified key clusters and pathways as well as specific genes (SEC61β, SLA7) that play important roles in animal health. Finally, the up regulation of IL1β in Duroc could explain the better immune response of Duroc when compared to Pietrain [66].

#### 3.2.2. Bone Marrow Derived DC

Mateu’s group performed the first study in bone marrow derived DC (BMDC), which extensively used BMDC to study PRRSV pathogenesis. They tested 39 European isolates that were able to induce different patterns of IL10 and TNFα production and different surface markers regulation. BMDC were productively infected by PRRSV isolated and MHCII upregulation was observed in selected PRRSV-1 strains [74]. Between the PRRSV-1 strains, high pathogenic Lena, together with Belgium A and Lelystad, reflected a different pattern. Lena showed a higher replication rate and apoptosis in BMDC when compared with other PRRSV-1 strains (Lelystad and Belgium A), but controversially it induced SLAII down regulation together with CD14, SWC3, and CD163 [75].

A most recent publication from Mateu’s group had characterized, in more detail, the interaction between PRRSV-1 strains and BMDC [76] when PRRSV-1 replication and attachment in immature (iBMDC) and mature BMDC (mBMDC) was studied. Replication kinetics showed that titres in iBMDC were significantly higher than mBMDC by 24 hpi and iBMDC were more efficient in the support of PRRSV-1 replication than mBMDC. iBMDC attachment by all of the strain was possible in cells that lack porcine CD163 or sialoadhesin (CD169) receptors or in cells with heparan sulfate (unspecific attachment receptor) removed. PRRSV-1 nucleocapsid could be observed in CD163− iBMDC and those cells were only infected when CD163low/hi cells were present, indicating that the susceptibility of CD163− cells derived as result of the milieu that was created by CD163+ infected BMDC, by receptor-independent mechanisms or that some cells expressed CD163 at levels that were below the technical sensitivity [76].

This study, together with most recent ones [64,77,78], questioned the notion of CD163 relevance during the infection. Nevertheless, the essential role of this receptor for viral uncoating and pathogenesis is still supported by the generation of genome edited pigs that lack the CD163 SRCR5 domain, which showed to be resistant to PRRSV infection [16,17].

As a whole, DC responses against PPRSV showed a general dysregulation of the IFN response, downregulation of activation and maturation markers with an induction of IL10 and Treg (Figure 1 and Figure 2).

#### 3.2.3. Plasmacytoid DC

Plasmacytoid dendritic cells (pDC) are the major source of type I IFNs and other inflammatory cytokines after exposure to viruses. Type I interferons are essential for direct antiviral activity and, despite pDC low frequency, they can produce around 100 times more IFNα than any other cell type and sense viruses in the absence of replication [79]. In pigs, these cells (Table 1) represent 0.1–0.3% of blood leukocytes and their role during PRRS was not clear until 2010 when few groups started to consider the involvement of these cells during the infection.

Zuckermann’s group studied, for the first time, the exposure of pDC, much broadly defined as CD4hi CD172alow CD1a+ CD11a+ CD11b− CD11c− CD16+ CD18+ CD29+ CD44+ SLAII+, to several pig viruses and, among them, American PRRSV [80]. PRRSV-2 46448 was not able to induce the production of IFNα in pDCs, even when doses were 100-fold. PRRSV-2 was able to stimulate a low but detectable IL2 production, but it failed to induce detectable IL8, IFNγ, TNFα, IL12, and IRF7 production. PRRSV exposed pDC remained relatively inert, showing unaltered morphology and CD80/86 downregulation when compared with untreated cells [80]. Subsequently, the study was expanded with several PRRSV-2 strains that were used in combination with potent pDC stimulators as TGEV and ODN D19 [81]. Interestingly, prolonged incubation of porcine pDC with PRRSV-2 did not significantly alter cell viability and pDC were resistant to the infection. Additionally, pDC suppression occurred independent of virus viability and the acidification of pDC early endosomes, but correlated with diminished levels of IFNα mRNA. This change was attributed to an abrogation of transcription resulting from a decrease in IRF7 production, limited as a consequence of the nuclear translocation block of STAT1. PRRSV strains confirmed TNFα synthesis inhibition but promoted NFkB phosporilation, which is necessary for pro-inflammatory cytokines expression [81].

Zuckermann opened a new research direction in PRRSV that was subsequently taken over by the Summerfield group in the last five years. They started to explore interactions of both PRRSV genotypes with pDC using a broad spectrum of PRRSV-1 and 2 strains [82,83]. Controversially, Summerfield at al. demonstrated how several type 2 strains induced weak or no suppression of IFNα in CpG-stimulated pDC and stimulated IFN-α in CD172alow CD4hi CD14− pDC. Interestingly, a high percentage of pDC was observed after PRRSV stimulation when compared to mock, suggesting the promotion of pDC survival by the virus [82]. Additionally, in this study, PRRSV sensing by pDC did not require live virus and pDC were confirmed to not be permissive to PRRSV. IFNα response involved the activation of the TLR7 pathway and it was enhanced by IFNγ and IL4. A surprising finding was that moDC were protected from PRRSV infection and killing when cultured with enriched pDC [82]. The divergent outcomes that were obtained by Zuckermann and Summerfield may lead back to the different pig genetics and to the diverse pDC isolation method and virus strains that were adopted in the studies. The role of pig genetics in the different outcomes after PRRSV infection has been reported [84,85,86], but its relationship with innate immune responses and particularly with pDC remains to be elucidated [87].

Data from both groups and genotypes found agreement only when pDC were exposed to Lena, the recent virulent PRRSV-1.3 strain that showed not to be able to induce IFNα in pDC in vitro but pig infected in vivo with Lena showed a systemic IFNα response [83]. In particular, an exosome fraction of Lena-infected cells but not Lena virions themselves were able to activate pDC [83].

PRRSV infected macrophages were more potent in activating pDC independently of the viral strains. This activation required cell adhesion molecules mediating contacts between MΦ and pDC, intact cytoskeleton and sphingomyelinase activity, but it was not induced by free PRRSV virions released from infected macrophages. Additionally, ITGAL-mediated intercellular adhesion was required for efficient sensing of PRRSV-infected MΦ [83].

Taken together, all of the findings demonstrate that pDC respond to PRRSV-1 and 2 genotypes and suppressive activities are moderate and strain-dependent (Figure 1). They may be a source of IFNα responses reported in PRRSV-infected animals, further contributing to the puzzling immunopathogenesis of PRRS.

### 3.3. Neutrophils

Pigs that develop interstitial pneumonia in the lungs after PRRSV infection normally show the mononuclear infiltration of alveolar septae and accumulations with macrophages and cell debris in the alveoli. Generally, high pathogenic strains exhibited severe pathology with increased neutrophils, mast cells, and macrophages when compared with low virulent strains [75,88]. Additionally, PRRSV-2 (IAF-Klop) infection leads to a significant increase in proteolytic activity in pulmonary fluids. Maximal activity was found at 7 and 14 days pi, with a return towards normal levels at day 42. Zymographic analyses showed a significant increase in the secretion of matrix metalloproteases 2 and 9, which are two enzymes involved in tissue remodeling [89].

Neutrophils (PMNs) interact with opsonized immune complexes through Fcγ receptors, activating and inhibitory receptors, which bind the Fc domain of IgG. In a study using PRRSV-2 HN07-1 or BJ-4, viral infection downregulates PMNs antibody-dependent phagocytosis and also impaired PMNs ability to kill *E. coli*, thus confirming that PMNs were impaired during PRRSV infection. In infected animals, the expression of FcγRIIIA inhibitory receptor decreased and reached the lowest point at 5 dpi in both PRRSV strains, and together with the late upregulation of FcγRIIIB, both contribute to decreased PMNs phagocytosis. The oxygen burst function of the PMNs was also depressed, and generally the consequences of infection by the more pathogenic strain HN07-1 were greater [90].

In another study, the PMNs infiltration was determined by the measurement of myeloperoxidase and enzyme activity in the lung, together with qPCR. In the lung, IL8, which is chemoattractant for neutrophil recruitment, was upregulated, and ICAM-1, responsible for firm neutrophils adhesion and transendothelial migration, was high in naturally infected animals. Moreover, VCAM-1 displayed a high level in experimentally and naturally infected pig lungs [91]. The induction of IL8 by PRRSV was further confirmed in vivo and in vitro, and it is likely through the TAK-1/JNK/AP-1 pathway [92].

In summary, PRRSV infection mainly impairs PMNs antibody-dependent phagocytosis and bacteria killing ability, together with the depression of the oxygen burst function, but seem to induce IL8 production (Figure 1 and Figure 2).

### 3.4. NK and γδ Tcells

It is surprising that very few works have studied NK and γδT cells during PRRSV infection. One of the first preliminary studies was performed in Spain, where the piglets were inoculated with PRRSV-1 5710. T cell cultures that were established by stimulating responding cells with PRRSV showed an increase of double positive memory CD8+CD4+ as well as CD4−CD8+ effector lymphocyte subsets within activated cells, whereas CD4+CD8− declined along the time. Within the activated cells, those expressing the TCRγδ receptor also increased, with most of them also being positive for CD8. In resting cells, the majority of γδ cells were CD8− [93]. Almost concomitant, the Bianchi’s group studied the change in detailed CD8+ cells subpopulations in BAL fluid in pigs that were infected with PRRSV-1 TerHuurne. NK (Table 1) were the main cells present in the lung of gnotobiotic and SPF piglets during the first days of infection, whereas CD8+ γδ T cells presence was never relevant After day 7, the increase of CD8+ cells correlated with a rapid decrease of PRRSV in the BAL fluid and CD8+ γδ T cells disappeared in the CD8+ cells [94]. Nevertheless, the use of different European strains showed different patterns in BAL leucocyte populations at early and late time points. In fact, at day 3 pi a significantly higher percentage of CD8− γδ T cells was observed in pigs that were infected with Belgium A and Lelystad, but not in strain Lena. At day 35 pi, cytotoxic T cells were almost double in percentage in all infections and CD8− γδ T cells were significantly lower [95].

On the other side, in a Canadian study using a PRRSV-2 experimental infection (LHVA-93-3), Magar’s group investigated the persistence of the virus in blood, spleen, lymph nodes, and tonsil. The authors discriminated between different CD8+ T cell subsets, and also between those NK cells. They defined NK cells as CD2+ CD8low and MIL4+, and they were not significantly modified in spleen and blood during infection in spite of a transient increase in the spleen at 3pi, followed by a gradual decrease up until 60 days pi. However, NK cells were rarely present in the tonsil and mediastinal lymph node, and they increased only at 3 days pi. Thus, it seemed that NK were not significantly modified during PRRSV-2 infection [96].

With the prototype VR-2332 in germ free piglets, the proportion γδT cells and NK decreased in BAL and only the CD2+CD8a+ γδ T subset increased. Tracheo-bronchial and mesenteric lymph nodes showed no differences in frequencies of NK and γδ T, but the CD2+CD8α+ subset increased together with a proportional decrease in the CD2-CD8α− subset [97].

An interesting study was performed in gilts, which were experimentally inoculated twice with PRRSV-2 MN-30100 and monitored for lymphocyte subpopulations, antigen specific proliferation, and IFNγ production. Following primary exposure to PRRSV, peripheral circulating γδ T cells percentage increased from day 14 to day 70, and then decreased to control at 120 days. γδ T cells responded to PRRSV infection significantly when compared to CD4 at an early stage and they were the major producer of IFNγ throughout the study [98]. Another study using different Minnesota strains, together with the prototype PRRSV-2 VR2332, showed an opposite outcome in tissues (PBMC, lung, tonsil, LN, bone marrow, spleen). PRRSV infection did not change the CD4+ or CD8+ population in any tissue, and by contrast, the γδ T cells were significantly decreased in lung and all LN and reduced non-significantly in every other tissue [99].

Seven-week-old nursery pigs in a commercial setting were injected with MN 1-18-2, and at day 2 pi, approximately 50% of viremic pigs had greater than 50% reduction in NK cell mediated cytotoxicity. Reduced frequency of CD4−CD8+ and CD4+CD8+ T cells and upregulated frequency of lymphocytes bearing natural Treg phenotype was detected in viremic pigs. All of the viremic contact pigs also had comparable immune cell modulation [100].

More recently, the interaction between NK and PRRSV-infected PAM was investigated in vitro. NK cytotoxicity assay was performed while using enriched NK cells as effector cells and Lelystad PRRSV-1-infected PAM as target. NK cytotoxicity against PRRSV-infected PAM decreased, starting from 6 hpi till 12 hpi. UV inactivated PRRSV also suppressed NK activity, but much less than infectious PRRSV, and co-incubation with infected PAM inhibited the degranulation of NK cells. By using supernatant from infected PAM, data showed that the suppressive effect of PRRSV in NK cytotoxicity was not mediated by soluble factors [101]. Successively, Cao et al. still considered the involvement of NK and γδ T cells during a vaccination study with a recombinant MLV vaccine that was incorporated with the porcine IL15 or IL18 gene fused to a signal that can anchor the cytokines to the cell membrane. In this case, immunization enhanced NK and γδ T cells responses and conferred improved protection against heterologous challenge (NADC20) [102].

Even with a limited number of studies, the data indicated that NK and γδ T cells interaction during PRRSV infection is altered with a suppressive effect on NK and the modulation of γδ T cells during the course of the infection. In particular, this is relevant when considering that γδ T cells are an important source of IFNγ during PRRSV infection (Figure 1 and Figure 2).

## 4. Innate Cellular Immune Responses Triggered by SwIV

### 4.1. Macrophages and Dendritic Cells

It is worth mentioning some in vitro experiments by several groups that attempted to understand the interaction of these cells with SwIVs. Kim et al. (2009) showed the MΦ culture supernatant from MΦ infected with SwIV H1N2. Significant differences in TNFα concentration between SwIV-infected and uninfected alveolar MΦ were detected at different hpi, with a peak at 36 hpi. These results suggested that TNFα might be an important mediator in the pathophysiology of SwIV infection [103].

Another in vitro study used three-dimensional/four (3D/4) cells, a spontaneously transformed line of swine MΦ (ATCC), infected with a pandemic H1N1 virus [104]. This report demonstrated that A (H1N1)pdm/2009 retains the ability to infect and replicate in swine MΦ, inducing a typical cytopathic effect (16 h pi) and destroying the cell monolayer (32 h pi). This study also examined the pattern of cytokine responses in pH1N1-infected swine MΦ by real time RT-PCR. IL6 and IL8 levels were up regulated at 16 h and the level of IL8 continued to rise up at 36 hpi. The robust induction of antiviral IFNβ and TNF family members, which may be attributable to cell death, was also observed. FasL and TNFα remained undetectable, while the TNF-related apoptosis-inducing ligand (TRAIL) seemed to be the most abundant one before infection. FasL and TNFα were most robustly induced, but TRAIL was only mildly induced in response to infection. The level of IL1β remained unchanged throughout the infection (different from Barbe et al. [105]), indicating that IL6 and IL8, as well as TNFα, were the main pro-inflammatory cytokines that were up-regulated. The authors also observed the induction of RIG-1 and MDA-5, which appeared to be completely suppressed by inhibitors of ERK1/2 or JNK1/2. This indicated that the induction of RIG-1 or MDA-5 depends on the activation of ERK1/2 and JNK1/2 in pig MΦ [104].

For the first time, our group described the interaction between porcine bone marrow-derived DC (poBMDC) (cDC) and SwIV H3N2 in vitro. The infection of poBMDC resulted in a structure resembling IV inside vesicles and also free in the cytoplasm of the cells. Viral progeny was undetectable in the supernatant but limited replication was detected in the first 8 hpi. However, the viral particles from infected-poBMDC were only able to induce a cytopathic effect in susceptible cells when cell-to-cell interaction was favored [106]. Additionally, they observed that similarly to the SwIV H3N2, porcine DC also supported a limited replication of other IVs during the first 8 hpi, without release of infectious progeny [106]. Additionally, these viruses similarly modulated the expression of NFκB, TGFβ and IL10 genes. However, they induced different kinetics and levels of inflammatory cytokines. Infection of poBMDC with SwIV induced a peak of IFNα secretion at 24 hpi, whereas, with the others, the production of IFNα was not detected. SwIV and highly pathogenic avian influenza (HPAI) induced more TNFα when compared to huIV and low pathogenic avian influenza (LPAI). SwIV, LPAI, and HPAI induced an increase of IL12 from 16 to 24 hpi and all of the viruses used induced IL18 secretion in a time-dependent manner [107].

Summerfield’s group also used GM-CSF derived DC infected with other avian and porcine IVs. They also generated recombinant reassortants by reverse genetics to elucidate the role of the single gene segments in the activation of cDC. The highest IFN type I responses were achieved by porcine virus reassortants that contained the avian polymerase gene PB2. This finding was not due to the differential tropism, since all of the viruses infected GM-CSF derived DC equally (and also porcine PK-15 epithelial cells) and infectivity was independent of HA expressed by the virus. All of the viruses induced MHCII, but porcine H1N1 expressing the avian viral PB2 more prominently induced nuclear NFkB translocation when compared to its parental strains. Therefore, in the case of porcine DC, PB2 was defined as an important viral element controlling IFN type I. While all the viruses had a comparable ability to infect DC, to initiate replication and to activate the cells in terms of MHCII induction, only those expressing PB2 derived from H5N1 were unable to prevent IFN type I induction; however, no viral progeny was detected [108].

When considering the distinct features of pDC and the crucial role of IFN in fighting virus infections, Bel at al. [109] analyzed the interactions of different influenza A viruses isolated from avian, human and swine with pDC obtained from pigs. Their results demonstrated that porcine pDC could produce high levels of IFNα in response to all of the tested strains, with subtype-specific differences in a virus-dose dependent manner. High levels of IFNα were detected upon live, chemically inactivated, or UV-inactivated virus stimulation. In contrast, heat-inactivated virus failed to induce a response. The observation that chemical and UV-inactivation did not abolish IFNα release indicated that non-infectious particles are also stimulatory for pDC. Additionally, these treatments did not abolish hemagglutination, suggesting that the integrity of HA and its binding function are necessary in inducing IFNα responses in pDC. At low viral doses, H5N1 and H7N1 avian viruses are more efficient in infecting pDC when compared to human H1N1 and at inducing the secretion of IFNα [109].

In vivo infection with SwIV mainly occurs in the respiratory-tract and the lungs of infected pigs, thus innate cells in the lungs are the first ones encountering the virus. One of the main components of the respiratory immune system is the DC/MΦ network that is involved in sensing foreign antigens, controlling inflammation, and initiating the adaptive immune responses. We have adapted a recently proposed nomenclature from Guilliams et al. [110] distinguishing between two levels of identification. The first level focuses on the origin of the cell-type progenitor (adult bone-marrow proDC for conventional DC (cDC), adult blood monocytes for monocyte-derived cells (moCells), or embryonic monocyte-derived precursors that were settled in peripheral tissues for MΦs. The second level focuses on the cell functions (MΦ-like or DC-like). We have recently finely defined the phenotypes and functions of DC/MΦ populations in the different compartments of the swine respiratory tract at a steady state and upon IAV infections in the pig [31]. We have defined six populations within the MΦ-DC network in pig trachea and lungs: 1—Porcine alveolar MΦ (AM) CD163hi/CD11b-likeneg and expressed high levels of MerTK and CD64; 2—AM-like population, the CD163hi ‘interstitial’ AM, unambiguously localized in the interstitium and representing >50% of the SLAIIhi parenchymal cells; 3—A third MΦ-like cell was described as SLAIIhi lung population: the CD163int cells. According to their MΦ features and their CCR2 and CX3CR1 expressions, they can be considered as moMΦ; 4—inflammatory moDC CD163low; 5-cDC1, FLT3, CD172a− expressing XCR1; cDC2, CD163−/CD172a+/XCR1−/Langerin+. After the infection of pigs with two field isolates, the CD163low/moDC population was the only one that significantly increased in numbers after both swine (sw)H3N2 and swH1N2 infections. Sorted lung CD172a−/cDC1 produced more IL12A mRNA, the Th1 inducer cytokine, than CD163−/cDC1 and CD163low/moDC, both in mock and IAV-infected animals in agreement with their Th1-inducing capacities in allogeneic reactions. Neither IL13 nor IL4 transcripts, which are the Th2-inducing cytokines, were detected. Finally, no differences in IL6 transcription were observed between the three DC subsets, both at steady state and upon infections, which is in agreement with the absence of a specific allogenic Th17-inducing DC subset [31].

Recently, a study highlighted the role of the inflammasome activation within influenza virus infection, in which a 2009 pandemic H1N1 induced less IL1β than swine influenza viruses (SwIVs). Their in vitro studies revealed that the NS1 C terminus of pandemic H1N1, but not that of SwIV was able to significantly inhibit NLRP3 inflammasome-mediated IL1β production, revealing a new mechanism of innate immune evasion achieved by the NS1 protein in pH1N1/09 [111].

**Figure 3:**
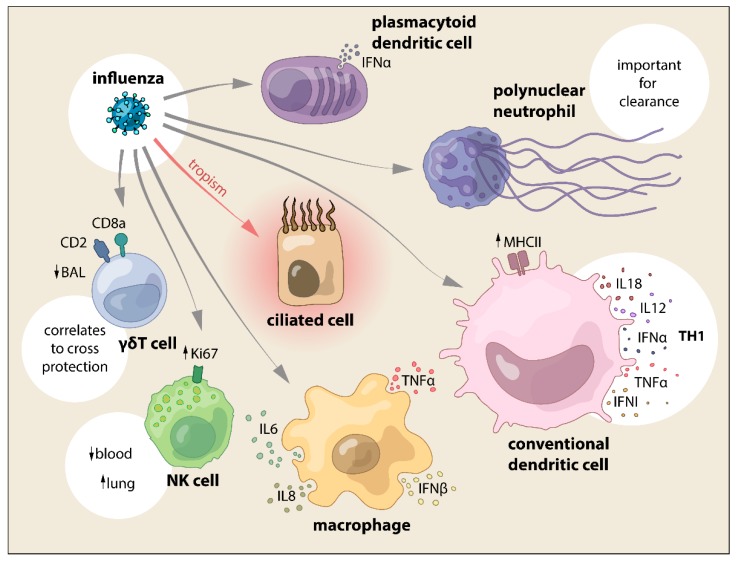
SwIV interaction with cells from the innate immune system and the main effects reported.

In summary, data showed that SwIV interacts with DC in vitro by inducing different kinetics and levels of inflammatory cytokines. Porcine pDC can produce high levels of IFNα, with subtype-specific differences in a virus-dose dependent manner, with PB2 being an important control factor for IFNα secretion in pDC. However, in vivo only CD163low/moDC population significantly increased in numbers after SwIV and lung CD172a−/cDC1 produced more IL12A mRNA than their counterparts, showing a distinctive activation pattern (Figure 2 and Figure 3).

### 4.2. Neutrophils

Neutrophils are important host defense cells against influenza virus during the phase of innate immunity. Although the role of neutrophils against influenza virus infections has been debated, neutrophils have been shown to play a role in the control and clearance of the influenza virus in experimental models [112,113,114,115].

The results from the study from Kim et al. demonstrated SwIV nucleic acid that is present in neutrophils by in situ hybridization yet, it was not clear whether the SwIV virus could replicate in the porcine neutrophil although the human influenza virus can replicate in human neutrophils [116]. One important function of neutrophils is phagocytosis. Hence, the SwIV that was detected in the neutrophils may have resulted from uptake (Figure 3). Further study needs to determine whether SwIV can replicate in porcine neutrophils and their role in influenza infection in the pig, as the role of neutrophils in SwIV infection is far from understood (Figure 2 and Figure 3).

### 4.3. NK and γδ T

Upon binding to the influenza virus, HA, the receptors trigger the NK cells to lyse the infected cell [117]. It has been suggested that invariant NKT (iNKT) cells stimulate the induction of cellular immunity and regulate infection-induced pathology [118]. With the identification of specific NK cell markers in pigs [42], some studies regarding the role of these cells in influenza infected pigs have been addressed. However, there are still few reports on the role of NK cells in swine during influenza virus infection.

Recently, it was demonstrated that NKp46+ lymphocytes accumulate in the vicinity of influenza A-infected cells in the lung of infected pigs. NKp46+NKcells are recruited from the blood to infected parts of the lungs in swine that were inoculated with the 2009 pandemic influenza virus at the same time as a decline in NKp46+ NK cells was demonstrated in the blood of infected pigs. Moreover, influenza virus does not infect the NKp46+ cells in the lungs and they do not undergo apoptosis [119].

Another study investigated the possibility that, besides NKp46+ NK cells, CD3+NKp46+ cells might also be involved in influenza infection. Thus, this lymphocyte population was analyzed in animals that were experimentally infected with the 2009 pandemic H1N1 influenza virus strain. A significant decrease in the total number of CD3+NKp46+ lymphocytes in PBMC could be observed in infected animals 1 dpi when compared with control animals. Furthermore, a significant increase in the frequency of CD3+NKp46+ lymphocytes could be detected in the lungs of infected animals as compared with the control group on day 3 pi. When compared with analyses from day 1 pi, a significant increase in proliferation by Ki67+ cells could be detected within CD3+NKp46+ cells in the lungs on day 3 pi [43]. Although anti-NKp46 mAb has been used to define NK during SwIV studies, the same approach has not been considered during PRRSV infection studies.

Likewise, few studies have been performed on the role of porcine γδ T cells during swine influenza infection. It has been shown that this subset increases in the lung following infection with H1N1 virus [120], in particular, the levels of γδ T cells were significantly higher in BAL and lower in tonsils of infected pigs when compared with control. Conversely, in another previous work, the γδ T cells percentage in the lung remained unchanged during H3N2 and H1N1 infections [121], showing a marked discrepancy in the results.

During a vaccination study, the level of γδ T cells was considered in cross-protection. A reverse genetics-derived H3N2 SwIV with truncated NS1 and wild type viruses were used to evaluate T cell priming and cross-protective efficacy against heterosubtypic H1N1 challenge. In control animals that were challenged with H1N1, there were no changes in the γδ T populations along the experiment, whereas immunization induces antigen-specific γδ T cells, including IFNγ and IL10 recall responses, before and after heterologous challenge. The group with the most robust γδ T cell responses correlated with the greatest cross-protection, suggesting that these cells may have had a protective role during the infection [122].

Finally, in a comparative study with PRRSV infection, using H1N1 SwIV in germ free piglets, the different subpopulation of NK and γδ T cells were evaluated. In the BAL of infected pigs, a decrease in the proportion of γδ T cells or NK cells was shown. The CD2+CD8a− γδ T subset was comparable to control animals, whereas CD2+CD8a+ γδ T increased, and CD2−CD8a− γδ T subset was lower than the control. In the tracheobronchial draining lymph node, there were no differences in the frequencies of NK and γδ T, but the distributions of the CD2+CD8α− subset increased. Similarly, the mesenteric lymph node analysis of γδ T cell subpopulations revealed no significant change in the proportion of any subset [97].

The few studies on the role of NK and γδ T cells during SwIV infections show that they may have a relevant function during infection and clearance, yet their interaction is far from understood (Figure 2 and Figure 3).

## 5. Innate Immune Responses Triggered by SwIV and PRRSV Co-Infection

While considering PRRSV and influenza virus co-infection, it is important to point out that both of the viruses have a different cell tropism, with macrophages being the main target of PRRSV and respiratory epithelial cells the main target of SwIV. Taking into account these premises, very few studies considered PRRSV-SwIV co-infection at the molecular level in the experimental set up, although both of the viruses are relevant contributors during PRDC and lung infections [1,123,124], and their presence is frequently observed during serological studies under field conditions [4].

Epidemiological studies have been performed to construct statistical models to evaluate a significant association between PRRSV and SwIV or other co-infectious agents, and to assess the effects of changes in age and management system on coinfection status, serological profiles, lung lesions, and histological lesions. One study was performed in piglets with different ages and logistic regression models were used to assess the co-infection. Clinically ill PRRSV-positive pigs were more likely than PRRSV-negative pigs to be co-infected with SwIV and to have lung scores that were in the 11 to 50% range. Nine and 16-week old pigs were 15.57 and 5.75 times as likely to be co-infected with SwIV, respectively [125]. Similarly, a statistically important association between pre-weaning infection with SwIV and PRRSV and post-weaning mortality was detected, with the season and number of days on feed also being associated [126]. US seroprevalence of PRRSV and SwIV co-infection in finisher herds was also estimated by the USDA NAHMS swine 2000 national study, with 4.9% herds serologically positive and 5.9% finishers (regardless the vaccination status) [127].

Early studies during the 1990s were mainly related to clinical and histopathological findings, where the inflammation of the bronchiolar wall was more pronounced in PRRSV/SwIV infected pigs than PRRSV, bronchiolar and lung lymph nodes were larger in the co-infection than in SwIV alone, but, at the end the PRRSV infection did not aggravate the acute stage of SwIV [128], or SwIV was only slightly affected by prior PRRSV [129].

Only in the last five years, Meurens’ group has been the one primarily investigating the interaction between SwIV and PRRSV in an experimental in vitro system. The first study was performed in porcine alveolar macrophages (PAM) and precision cut lung slices (PCLS) from eight-week-old pigs. They used the PRRSV-2 VR-2385 and SwIV Canadian strain H1N1 applied simultaneously or 3 h apart on PAM and PCLS for 18 h. Interference that was caused by the first virus on replication of the second was observed and a synergic effect between PRRSV and SwIV was observed for some transcripts, such as TLR3, RIG1, and IFNβ in PCLS. PRRSV infection 3 h prior SwIV reduced the response to SwIV, while the SwIV infection prior to PRRSV infection had limited impact [130]. The second recent study was performed in a trachea epithelial cell line expressing CD163 (NPTr-CD163), which is the main receptor for PRRSV. The cell line was receptive to both viruses and was used to assess the interference between the two. SwIV and PRRSV interacted differently with the modified cell line, but they were interfering each other in terms of replication when infected in the same cell with consequence on the antiviral response (LGP2, MDA5, TLR8, IFNα, IFNβ, IFNλ1, MX2, OAS, PKR) [131].

More studies are required to precisely define the interaction of both viruses on the immune system and the consequences for disease and vaccination.

## 6. Conclusions

Given the importance of the innate immune system during the first critical hours and days of exposure to a new pathogen, it is surprising to realize how scattered the information is when speaking about two of the main players in PRDC. PRRSV interaction with MΦ and DC has been studied in some detail, but there are controversial data between different groups. For SwIV, the picture seems more in agreement, but the whole picture of fine-tuning mechanisms of virus interaction with the host innate immune is still far from complete. The modulation of NK, PMN, or γδ T cells, in general, has not received as much attention as their APC counterparts and there are numerous gaps in the knowledge regarding the role of these cells in both virus infections and their interaction between them. Finally, studies on the co-infection of PRRSV-SwIV have received little attention within the scientific community, and even epidemiological studies have shown significant association between PRRSV and SwIV. Studies in these directions will pave the way to understand PRDC in better detail and possible design strategies to combat this disease.

## Figures and Tables

**Table 1:** Porcine innate immune cells phenotype and porcine reproductive and respiratory syndrome virus (PRRSV) and swine influenza viruses (SwIV) susceptibility.

Porcine Innate Immune Cells	Phenotype	PRRSV Susceptibility	SwIV Susceptibility
MoDC BMDC cDC 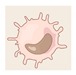	*In vitro* moDC and BMDC SLAII+, SLAI+, CD80/86low/+, CD16+, CD14low, CD172a+, CD1+	Yes	Limited replicationin BMDC *(Mussa et al. 2011)* Replication in GM-CSF derived DC *(Ocana-Macchi et al. 2012)*
	*In vivo* tracheal cDC1 and cDC2 (*Resendiz et al. 2018*) cDC1, CD163-, SLAIIhi, CADM1+, CD172a −, FLT3+, XCR1+ cDC2 CD163-, SLAIIhi, CADM1hi, CD172a+, FLT3+, FcεR1α+	No	N/A
	*In vivo* lung DC (density gradient separation and CD11c+) (*Loving et al. 2007)* SLAI+, CD80/86+, SLAII+, and CD16+, CD14low, CD172a+, CD1+	No	N/A
	*In vivo* lung DC (*Proll et al. 2017*) CD11c+, CD86+, CD80+, CD40+	Yes	N/A
	*In vivo* lung CDC1, CDC2, moDC (*Bordet et al. 2018*) cDC1 SLAIIhi, CD163-, CD172a-/low, CD11c+, CadM1+ XCR1+ cDC2 SLAIIhi, CD163-, CD172a+, CD11c+, Cadm1+, CD1+ FcεRIα+ moDC SLAIIhi, CD163low, CD172a+, CD11chi	No	*In vivo* moDC increase in number during SwIV *(Maisonnasse et al. 2016)*
pDC 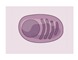	*In vivo* CD4+/hi, CD172alow/+, CD1a+, CD11a+, CD11b-, CD11c-, CD16+, CD18+, CD29+, CD44+, SLAII+, CD123+, CD135+, CD14-	No	N/A
Macrophages 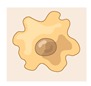	*In vivo* lung alveolar MΦ CD163+, CD169+, SWC9+ (CD203a), CD172a+, CD14+, CD16+, and SLAII+	Yes	*In vitro* transformed 3D/4 cells infected by H1N1 pdm 2009 *(Gao et al. 2012)*
	*In vivo* AM-like/PIM MΦ CD163+, CD169+, CD172a+, CD14+, CD16+, and SLAII+	Yes	
Neutrophils 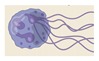	*In vivo* SWC1+ or CD21+, SWC8+	No	Not clear
NK and γδ T cells 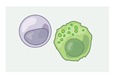	*In vivo* NK perforin+, CD2+, CD8α+, NKp46+, CD8β-, CD11b+, CD16+, CD3-, CD4-, CD5-/low, CD6-	No	No
	*In vivo* γδ T cells divided in 3 subsets: TCRγδ hi, CD2−CD8−, TCRγδ med CD2+CD8− and TCRγδ med CD2+CD8+	No	No
